# Melanocytic lesion in children and adolescents: an Italian observational study

**DOI:** 10.1038/s41598-020-65690-x

**Published:** 2020-05-25

**Authors:** Caterina Lanna, Chiara Tartaglia, Raffaele Dante Caposiena Caro, Sara Mazzilli, Alessandra Ventura, Luca Bianchi, Elena Campione, Laura Diluvio

**Affiliations:** 1 Dermatologic Unit, University of Rome Tor Vergata, Roma, Italy; 2 Dermatologic Unit, University of L’Aquila, L’Aquila, Italy

**Keywords:** Health care, Oncology, Risk factors

## Abstract

Malignant melanoma is a rare neoplasm in the pediatric age group. One of the main risks factors is represented by the presence of a high number of melanocytic nevi. Sun exposure in pediatric age represents a predictor of melanocytic nevi number in the adult age and there is a direct correlation between the presence of melanocytic moles in early childhood and the development of many nevi in adults, suggesting that a high number of nevi in childhood should be considered as a predictor of melanoma development during adult life. The predominance of dermoscopic types of melanocytic nevi varies according to the individual’s age and depends on endogenous or exogenous signaling, suggesting different pathways of nevogenesis. We evaluated the total amount of melanocytic nevi of pediatric patients and their prevalent dermoscopic pattern. We investigated the reasons for dermatological examination, pointing out the role of older parents’ populations in the decision to refer to a dermatological consultant. We performed a prospective observational study on 295 pediatric outpatients consecutively enrolled from July 2018 to July 2019. Descriptive and inferential statistical analyses were performed using logistic and linear regression. 49% of children were characterized by less than 10 nevi, 45% of children by a number of nevi between 10 and 30, whilst 17 patients (5%) had a number of nevi between 30 and 50. The most prevalent dermoscopic pattern was the globular one. An older parenting age was correlated with an autonomous reason for referral and a later first visit. Our data agreed with previous suggestions demonstrating a strong influence of latitude, sun exposure and ethnic background in the development of the number of nevi. To our knowledge, this is the first study, which evaluated the reasons for dermatological examination and the role of older parents’ populations in the decision to refer to a dermatological consultant

## Introduction

Malignant melanoma is a rare neoplasm in the pediatric age group, accounting for 1% to 4% of all cases of melanoma and for 1% to 3% of all childhood malignancies^1^, although the latest epidemiologic data from the Surveillance, Epidemiology, and End Results program (SEER) demonstrated an incidence rate of melanoma increasing by 2% per year in childhood^2^. As in the adult population, the pediatric risk factors include intermittent intense sun exposure, tendency to sunburn, tendency to freckle, fair skin, blue or green eyes, blond or red hair, xeroderma pigmentosum, giant congenital melanocytic nevus (CMN), immunosuppression and a family history of melanoma^3^. A genetic predisposition has been suggested in several studies that analyzed the amount of nevi in monozygotic twins versus dizygotic twins, with a stronger correlation in the monozygotic cases^4,5^. Perhaps one of the main risks factors for developing melanoma is represented by the presence of a large number of melanocytic nevi^6–8^. An increased risk has been found in adolescents with >100 nevi and >10 large nevi^9–11^. Moreover, as reported by Paradela, 50% of children with melanoma had sporadic dysplastic nevi, whilst 9% had dysplastic nevus syndrome^10^.The prevalence of nevi increased progressively with age and is associated to gender, skin type, ethnic origin and sun exposure^12^. It is well known that environmental factors play a pivotal role in the development of nevi and/or melanoma. The nevus count differs according to geographic locations, while sunlight represents the principal trigger element. Melanocytes are particularly predisposed to the biologic effects of sunlight during the critical period of early childhood^13^. Several pediatric studies demonstrated that the high number and early development of melanocytic lesions are correlated to the level of sun exposure^14^. Moreover, sun exposure in pediatric age represents a predictor of melanocytic nevi number in the adult age and there is a direct correlation between the presence of melanocytic moles in early childhood and the development of many nevi in adults^15^. Therefore, a high number of nevi in childhood should be considered as a predictor of melanoma development during adult life.

We reported the results of an observational study on 295 pediatric outpatients who referred to our department in one year.

We evaluated the total amount of melanocytic nevi showed by our pediatric patients and the relationship between that number and the patients’ sun exposure habits and protection. Moreover, we studied the influence of sunburns and/or positive family history of melanoma in the development of a higher quantity of nevi. Thus, we valued the most prevalent dermoscopic pattern showed by our pediatric population. We investigated the reasons for dermatological examination and pointed out the role of older parents’ populations in the decision to refer to a dermatological consultant.

## Methods

We performed a prospective observational study in the dermatologic department of Rome Tor Vergata General Hospital, Italy. We consecutively enrolled all pediatric outpatients from July 2018 to July 2019. Patients age varied between 15 months and 17 years. Detailed dermatological examinations were performed, including oral-genital mucosae, hair and nails.

Primary outcomes included: number of nevi, divided in three subgroups (<10 nevi, between 10 and 30 nevi, and between 30 and <50 nevi). Secondary outcomes included: most prevalent dermoscopic pattern (divided into three subgroups: globular, reticular and mixed), phototype, use of sunscreen, history of sunburns and a family history of melanoma. Parents were asked their age and the reason behind the dermatological examination (if it was a pediatrician’s referral or a parents’ decision).

The study was performed according to the principles of Helsinki Declaration and was approved by the institutional review board and ethics committee of the “Policlinico Tor Vergata” (Tor Vergata General Hospital). Written informed consent was obtained from the parents of each participant, while an interviewer completed a clinical questionnaire.

Descriptive and inferential statistical analyses were performed using logistic and linear regression.

## Results

A total of 295 pediatric outpatients were examined between 2018 and 2019 in our dermatologic department. 149 patients were males, 146 females. Mean age was 11.9 years old, ranging between 15 months and 17 years old.

**Figure 1:**
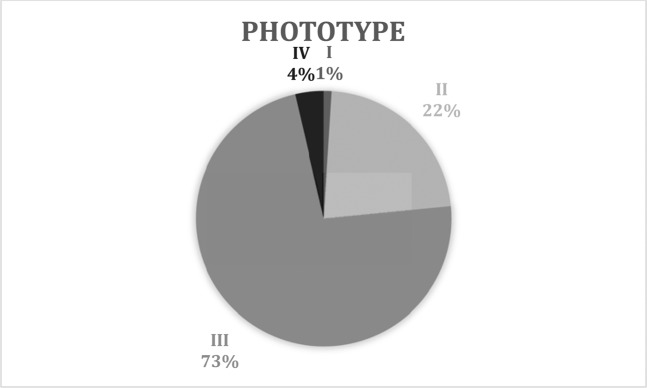
Phototype.

1 patient (1%) with phototype I, 66 (23%) with phototype II, 219 (74%) with phototype III, and 9 (3%) with phototype IV (Fig. 1).

**Figure 2:**
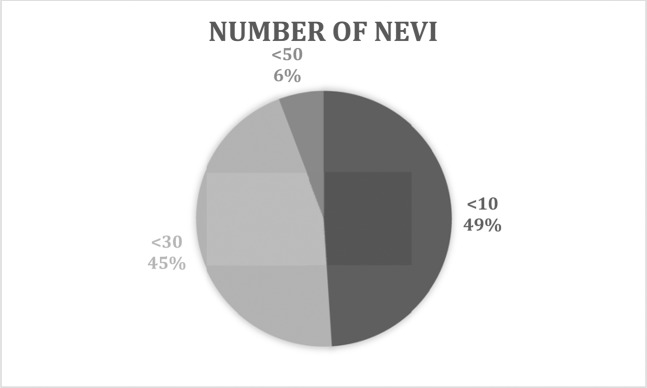
Number of Nevi.

145 (49%) children had less than 10 nevi, 133 (45%) children had a number of nevi between 10 and 30, whilst 17 patients (5%) had a number of nevi between 30 and 50 (Fig. 2).

5 of the 17 subjects affected by an amount of nevi between 30 and 50 were phototype II, whilst 11 were phototype III.

130 (44%) children were evaluated through epiluminescence microscopy every 12 months.

293 (99%) declared to use sunscreen protection, 21 (7%) patients reported a history of sunburns.

A family history of malignant melanoma was identified in 35 (12%) patients. One of those patients had a total of nevi between 30 and 50.

42 (14%) children were affected by atopic dermatitis or by other autoimmune diseases such as rhinitis, asthma, psoriasis, etc. No relation between a higher number of nevi and autoimmune diseases was observed in our study.

The most prevalent dermoscopic pattern was the globular one, present in 200 patients. Moreover, we evaluated that older children showed more reticular nevi while the younger ones had globular ones.

21 patients reported a history of sunburns, 18 of them had between 10 and 30 nevi, 3 of them had less than 10 nevi.

**Figure 3:**
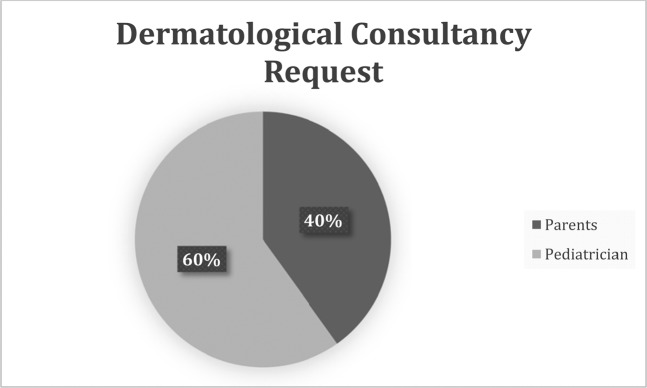
Dermatological Consultancy Request.

In 176 patients the referral to the dermatologist was chosen by the pediatrician (Fig. 3).

Both the analysis by regression and the correlation showed a statistical significance (p-value < 0.05) between the reason for the referral (parents’ vs. pediatrician’s decision) and the average age of the parents. Therefore, an older parenting age was correlated with an autonomous reason for referral, while younger parenting age was correlated with a pediatrician’s decision.

We also found a statistical significance between the age at first referral and the parents’ age (p-value < 0.05). Therefore, older parents corresponded to a later first visit.

## Discussion

This study investigated the amount of melanocytic nevi and factors that seem to be related to the incidence of these lesions in our pediatric dermatological population.

Most of our subjects had less than 10 nevi, which is less than several previously reported data such as the ones collected in Melbourne, Australia (mean melanocytic nevus count = 51.1), Sydney, Australia (66.5), Townsville, Australia (77.2), while it is similar to the mean melanocytic nevus count documented by Enta, Canada, (between 7 and 29) and by Rokuhara, Japan (2.5). In Turkey, Dogan *et al*., reported a mean of 1.1 lesion per person; in Switzerland, Sigg *et al*. counted 17.9 melanocytic nevi^14,16–19^. The Italian population was evaluated in 1990 by Colonna *et al*., reporting a mean melanocytic lesion of 8 nevi in children between 5 and 10 years of age^20^.

The higher incidence of melanocytic nevi in Australia could be explained by past migrations that caused a higher presence of phototype I or I-II in the Australian population. The exposition of these skin phototypes to Australian latitudes could have induced a greater development of nevi^21,22^.

Indeed, most of our patients showed skin phototype III, which seemed to be protective for the development of melanocytic nevi since only 11 subjects with this phototype developed between 30 and 50 nevi.

Therefore, our data agreed with previous suggestions, demonstrating a strong influence of latitude, sun exposure and the ethnic background in the development of the number of nevi^23^.

Almost all patients used sunscreens, demonstrating an increase in melanoma awareness and related risk factors. We explained this result considering an increased attention of mass media to melanoma and skin cancers.

We did not find any relationship between sunburns history and the amount of nevi, suggesting that latitude, sun exposure behavior and ethnic background - such as skin phototype - are more important for the development of melanocytic nevi. Indeed, intermittent sun exposure is considered a risk factor for moles development, while sunburns history is considered a risk factor for skin carcinomas^24^.

The most prevalent dermoscopic pattern was the globular one, present in 68% of patients, a result similar to that of previous studies^25^. Moreover, we found that older children showed more reticular nevi while the younger ones showed globular nevi, suggesting a different melanogenic mechanism related to different ages^25^. Indeed, our data may reflect the dynamic change in individual nevi over time.

A single nevus could initially show a globular pattern, which may evolve into a reticular and/or homogeneous pattern over time, although the actual transition of a globular pattern nevus into a reticular pattern nevus has not been documented yet.

However, our outcomes are in contrast with the notion of the natural evolution of nevi, which states that nevi begin as lentigo simplex, then change into junctional and later compound and dermal nevi^26^.

Nonetheless, Worret and Burgdorf found that children under the age of 10 showed purely junctional nevi, and often showed combined and dermal naevi^26^. Furthermore, Cramer pointed out the possibility that nevi can migrate from the dermis upwards into the epidermis^27^.

Additionally, Oliveria *et al*. found a globular dermoscopic pattern to be predominant, particularly in large nevi (diameter >4 mm), and speculated on whether this pattern could be read as a feature of “congenital-like” melanocytic nevi^28^. These observations suggest that nevi displaying a globular pattern might represent a subset of congenital melanocytic nevi of delayed (late) onset, in contrast to nevi rising after puberty and showing reticular and ⁄or homogeneous patterns. These nevi with reticular or homogeneous patterns might be acquired melanocytic nevi.

Lastly, there could be two independent types of nevi (congenital and acquired) among a population of different ages, which apparently follow two distinct pathways in their evolution. The congenital nevus develops until puberty and dermoscopically it is characterized by globular patterns. It persists in adults as a dermal nevus. It seems these congenital nevi respond to growth factors trough endogenous signaling. Instead, the acquired nevus seems to develop from puberty until 40–50 years old, disappearing/regressing after 60 years old. Dermoscopically, this nevus is characterized by reticular and⁄or homogeneous patterns with central hyperpigmentation. It develops in response to ultraviolet exposure (trough exogenous signaling)^25^.

For what concerns reasons for examinations, older parenting age was correlated with an autonomous reason for referral, while younger parenting age was correlated with decision by the pediatrician. We explained this aspect considering that older parents could have more experience, maybe due to previous experience with other children and do not need pediatrician indication for dermatological consultancy.

Indeed, older parents only requested dermatological consultancies for their children during adolescence, which could be explained by a better familiarity to medical and dermatological practice as well as knowledge of melanoma epidemiology.

However, later referrals could be also justified by less attention to dermatological diseases and less medical information.

In conclusion, our study assessed the role of sun exposure habits and protection and the influence of sunburns and/or positive family history of melanoma in the development of a higher quantity of nevi in a large cohort of children. Moreover, we pointed out the dermoscopic differences between younger children showing globular patterns and older children showing reticular patterns. Finally, to our knowledge this is the first study which evaluated the reasons behind a dermatological consultancy.

Further studies could better clarify nevus evolution and pathways responsible for nevogenesis. Ultimately, the correct classification of nevi and the discovery of risky habits for the development of a greater amount of nevi may help to better define populations with a higher risk to develop melanoma.

### Informed consent

Informed consent was obtained from all individual participants included in the study.
